# Validation of the Distress Thermometer as a Screening Tool for Psychosocial Distress and Resilience in Parkinson's Disease

**DOI:** 10.1002/mdc3.13937

**Published:** 2023-12-10

**Authors:** Nils Schnalke, Esther Tekampe, Tim Feige, Anika Frank, Heinz Reichmann, Björn Falkenburger, Simone D'Souza

**Affiliations:** ^1^ Department of Neurology University Hospital Carl Gustav Carus Dresden Dresden Germany; ^2^ Center for Neurodegenerative Diseases within the Helmholtz Association (DZNE) Dresden Germany

**Keywords:** Parkinson's disease, screening tool, distress, depression, anxiety

## Abstract

**Background:**

Parkinson's disease (PD) is associated with psychosocial distress that affects patients’ quality of life. The distress thermometer (DT) is an 11‐point visual analogue scale that is used as a screening tool for the assessment of psychosocial distress, originally developed for oncological diseases.

**Objectives:**

To validate the DT for PD and to explore contributing factors.

**Methods:**

The DT scale was administered to 105 people with Parkinson's Disease (PwPD). Along with it, we assessed motor symptoms (Unified Parkinson's Disease Rating Scale part III [UPDRS III], Hoehn and Yahr‐stage [H&Y]), non‐motor symptoms (Non‐motor Symptom Questionnaire [NMSQ]), anxiety and depression (Hospital Anxiety and Depression Scale [HADS], Fear of Progression‐Questionnaire Short Form [FOP‐Q‐SF], Generalized Anxiety Disorder Scale‐7 [GAD‐7], 9‐question Patient Health Questionnaire [PHQ‐9]), the feeling of hope (Herth Hope Index [HHI]) and quality of life (Schedule for the Evaluation of Individual Quality of Life [SEIQoL]).

**Results:**

With a cut‐off of 4, the DT identified PwPD with distress with a sensitivity of 97% and a specificity of 38%. With this cut‐off, the DT will yield false negative results in 1 out of 100 cases. Factor analyses and a random forest regression of the dataset revealed that distress can be predicted by two factors, which we termed “anxiety” and “depression/resilience/motor symptoms”.

**Conclusion:**

The DT is an ultra‐short and reliable screening tool for distress in PwPD. DT values below 4 rule out distress with a high degree of certainty. Anxiety and depression are important factors in distress but are counterbalanced by the individuals’ psychological resilience.

Parkinson's disease (PD) is a chronic and progressive disorder with a broad spectrum of symptoms. Motor symptoms in PD are readily treatable.^1^ Encouragingly, medical and non‐medical treatment strategies also exist for the various non‐motor symptoms, even if these are often more resistant to treatment.^2^

Distress is generally defined “as a multifactorial, unpleasant experience of a psychologic (ie, cognitive, behavioral, emotional), social, spiritual, and/or physical nature that may interfere with the ability to cope effectively with [the disease], its physical symptoms, and its treatment”.^3^ The concept of distress is therefore distinct from anxiety and depression, which can occur as non‐motor symptoms in PD. In various diseases and conditions, distress has been demonstrated to significantly affect quality of life and treatment outcomes.^3^

In oncology, distress is commonly assessed with the distress thermometer (DT),^4^ a single‐item screening instrument consisting of a visual analog scale developed by the Distress Management Guidelines Panel of the National Comprehensive Cancer Network (NCCN).^5^

The DT is typically administered in combination with a disease‐specific, predefined problem list (PL), which is not a part of this study.

The Hospital Anxiety and Depression Scale (HADS) has typically been used for DT validation.^6^ The most commonly identified DT cut‐off score is four.^3^, ^6^, ^7^, ^8^ Adapted and validated versions of the DT and PL are already being used to assess psychosocial distress in disciplines outside of oncology.^7^, ^9^

During preparation of this manuscript, a recent study demonstrated strong correlations of the DT with the Hospital Anxiety and Depression (HADS) subscales Anxiety (HADS‐A) and Depression (HADS‐D) in people with Parkinson's Disease (PwPD).^10^ However, a cut‐off score for the DT in PD has not yet been determined.

We therefore describe here our comprehensive validation of the DT in PD, which includes the definition of a cut‐off score for the DT, and an investigation of the factors that underlie distress.

## Methods

### Participants

This observational cross‐sectional study was conducted at the Department of Neurology at the University Hospital “Carl Gustav Carus” in Dresden, Germany, between January 2019 and October 2020. Inclusion criteria were (1) diagnosis of PD, (2) sufficient cognitive ability to complete the questionnaires according to the treating physician and (3) proficiency of written and spoken German. The study was approved by the ethics committee at Technische Universität Dresden (IRB00001473, EK 37012019) and conducted in accordance with relevant guidelines and regulations. We confirm that we have read the Journal's position on issues involved in ethical publication and affirm that this work is consistent with those guidelines. All patients gave written informed consent to participate in the study.

There are no accepted guidelines for calculating a sample size for validating patient reported outcome measures as pointed out by Anthoine et al.^11^ In general, a subject to item ratio of at least five to one is recommended for exploratory factor analysis, with an absolute minimum of 100 participants.^12^ Hence, we aimed for *N* = 100 datasets and enrolled *N* = 108 consecutive patients with Parkinson's disease in the waiting area of the PD outpatient clinic, where they waited for their regular follow‐up appointment. *N* = 3 participants were excluded: *N* = 1 misdiagnosis of essential tremor, *N* = 2 withdrawal of participation. Data of *N* = 105 participants were analyzed.

Patients were asked to complete questionnaires in the waiting room. Assistance from the caregivers or study team was acceptable if needed. *N* = 82 participants completed the questionnaire in the waiting room and *N* = 23 participants returned the questionnaires via mail because there was insufficient time to complete the surveys.

### Assessments

Patients were provided with the Distress Thermometer (DT) for Parkinson's Disease, the Hospital Anxiety and Depression Scale (HADS), the Patient Health Questionnaire‐9 (PHQ‐9), the Non‐motor Symptom Questionnaire (NMSQ), the Fear of Progression Questionnaire‐Short Form (FOP‐Q‐SF), the Generalized Anxiety Disorder Scale‐7 (GAD‐7), the Herth Hope Index (HHI), the Schedule for the Evaluation of Individual Quality of Life (SEIQoL) and a demographic survey as paper‐based questionnaires. The demographic survey included gender, age, marital status, education, occupational status, and disease related questions such as duration of disease or medication.

PD motor‐subtype, H&Y‐stage and UPDRS III total scores were collected from the routine clinical assessment of the same day as the outpatient clinic visit. German versions were used for all questionnaires.

#### Distress Thermometer (DT)

For validation, the DT in its German version (2006) was used. It is an 11‐point visual analog scale, with a score of 0 representing “no distress” and a score of 10 representing “extreme distress” (see Fig. S1 for the German version of the DT, Fig. S2 for the English version of the DT).

#### Anxiety and Depression

The HADS consists of the HADS‐Anxiety (HADS‐A) and HADS‐Depression (HADS‐D) subscales, each containing seven questions.^13^ The total HADS score is an indicator of general distress and is commonly used as the gold standard for validating the DT.^6^, ^7^, ^14^, ^15^ In accordance with the literature, the total HADS cut‐off score for distress was set at 15^14^ and at eight for the HADS‐Anxiety and HADS‐Depression subscales.^16^ The PHQ‐9 is a short, well validated screening test for major depression consisting of nine questions in total.^17^

There is a validated short form of the Fear of Progression‐Questionnaire (FOP‐Q) which encompasses 12 instead of 43 items (FOP‐Q‐SF).^18^ Together with the Generalized Anxiety Disorder‐7 (GAD‐7) questionnaire^19^ ^(p7)^, we used these assessments to further characterize symptoms of anxiety in our cohort of PwPD.

#### Assessment of Quality of Life (QoL)

To assess QoL in general, we used the Schedule for the Evaluation of Individual Qualitiy of Life (SEIQoL), which first asks about the importance of 12 different aspects of life to the individual person and in a second step inquires to which degree they feel satisfied with this aspect of their life. From this, a compound score can be calculated, which can be expressed as a life quality index on a scale from 0 to 100.^20^

### Statistical Analyses

Statistical analyses were performed using the SPSS version 27 (IBM, New York), Jeremey's Amazing Statistics Program (JASP 0.17.2, JASP Team 2023) and Jamovi Version 2.3 (The jamovi project 2022), the latter two are based on R (R Core Team 2022). A *p* value ≤0.05 was considered statistically significant in all analyses.

Statistical differences in subgroup analyses were computed by *t*‐Tests, Fisher's Exact Tests and *χ* ^2^ tests where appropriate. To assess convergent and discriminant validity, we calculated Pearson correlations of the DT score with the total HADS score, H&Y‐stage, and the NMSQ. Pearson correlations were calculated for all other study assessments as well. According to Cohen's r, *r* ≥ 0.3 was considered a medium effect size and *r* ≥ 0.5 a strong effect size.^21^, ^22^

The Receiver Operating Characteristics (ROC) curve with the total HADS cut‐off score of ≥15 was calculated and used to define the cut‐off score of the DT.^23^ The larger the area under the curve (AUC), the greater the discriminatory power,^24^ in this case the discriminatory power between distressed and non‐distressed PwPD. From this, the test properties (sensitivity, specificity, NPV etc.) were calculated. The prevalence of distress was set as the proportion of PwPD in our study who had a total HADS score of ≥15.

An exploratory factor analysis considering the total scores of all assessments was performed. For this analysis, z‐score normalization for all scores was applied, since the assessments have differing value ranges. Factors with an Eigenvalue of ≥1 were included, which is the traditionally applied Kaiser's criterion for factor analysis.^25^ To determine whether our data set is appropriate for this method, we calculated a Kaiser‐Meyer‐Olkin measure of sampling adequacy, which revealed an adequate sample size with a value of 0.879. Bartlett's test confirmed at least some correlation between the variables (*X* ^2^ test <0.001), which is a prerequisite for EFA. Since factors were highly correlated, an *oblimin* oblique rotation was chosen. Using this method, two factors were identified. We then further evaluated the factors identified by EFA by a confirmatory factor analysis and evaluated further fit indices. The same model was then used to train a random forest regressor to estimate DT scores from the total scores of the assessments contained in the two factors identified by the exploratory and confirmatory factor analyses.

## Results

Demographic and clinical characteristics of *n* = 105 patients enrolled in this study are displayed in Table 1. The mean DT score was 5.2 (standard deviation, SD 2.3).

**TABLE 1:** Demographic and Clinical Characteristics

Feature	*N* = 105	Mean (SD) or %
Gender, % male	68	65
Age (years)	102	66 (Range 35–87)
PD subtype		
Equivalent–type	31	29.5
Tremor–dominant type	25	23.8
Hypokinetic–rigid type	46	43.8
NOS	2	1.9
Missing	1	1
Hoehn & Yahr stage		
Hoehn & Yahr stage I	8	7.6
Hoehn & Yahr stage II	51	48.6
Hoehn & Yahr stage III	38	36.2
Hoehn & Yahr stage IV	5	4.8
Hoehn & Yahr stage V	0	0
Missing	3	2.9
UPDRS III total score	103	22.4 (10.3)
Disease duration [years]	105	8.9 (5.7)
Secondary diagnoses		
MCI	4	3.8
Dementia	6	5.7
Depression	18	17.1
Medication		
Levodopa	84	80
Dopamine agonist	78	74.3
COMT inhibitor	34	32.4
NMDA antagonist	14	13.3
MAO‐B inhibitor	47	44.8
Marital status		
Single	5	4.8
Married	82	87.1
Widowed	6	5.7
Divorced	10	9.5
Missing	2	1.9
Education		
School (≥ 10 years)	45	42.9
School (< 10 years)	19	18.1
University	36	34.3
Missing	5	4.8
Occupational status		
Working	27	25.7
Not working	77	73.3
Missing	1	1

Abbreviations: COMT, catechol‐o‐methyl‐transferase; MAO‐B, monoamine oxidase B; MCI, mild cognitive impairment; NMDA, *N*‐methyl‐d‐aspartate; NOS, not otherwise specified; PD, Parkinson's disease; SD, standard deviation.

To verify the construct validity of the DT, the convergent and discriminant validity of the DT was determined. To verify convergent validity, a correlation analysis was performed with the DT and the HADS and non‐motor symptoms as measured by the NMSQ. The moderate to strong correlations of the DT total score with the HADS total score (*r* = 0.472, *p* < 0.001), the HADS‐A (*r* = 0.504, *p* < 0.001), the HADS‐D (*r* = 0.379, *p* < 0.001) and the NMSQ score (*r* = 0.393, *p* < 0.001) indicates good convergent validity, that is, the DT is a similar construct in relation to these measures. To verify discriminant validity, a correlation analysis was conducted between the DT and H&Y‐stage. The DT has no significant correlation with H&Y‐stage and thus is conceptually independent.

Concerning the UPDRS III total scores (higher scores indicating worse motor function) as a measure of motor symptoms, these were only weakly correlated to the DT scores (*r* = 0.21, *p* < 0.05). UPDRS III scores were moderately negatively correlated with the HHI (*r* = −0.305, *p* < 0.01) and the SeiQoL (*r* = −0.319, *p* < 0.01) and positively correlated with symptoms of depression (HADS‐D *r* = 0.34, *p* < 0.01; PHQ‐9 *r* = 0.266, *p* < 0.01).

As expected, measures of anxiety were strongly positively correlated amongst each other (eg, FOP‐Q and HADS‐A *r* = 0.599, *p* < 0.001; GAD‐7 and HADS‐A *r* = 0.732, *p* < 0.001). Interestingly, measures of hope (HHI) and the life quality index as measured by the SeiQoL were strongly negatively correlated with the HADS (HHI and HADS *r* = −0.645, *p* < 0.001; SeiQoL and HADS *r* = −0.598, *p* < 0.001). These measures were also moderately negatively correlated to the DT score (DT and HHI *r* = −0.314, *p* < 0.001; DT and SeiQoL *r* = −0.354, *p* < 0.001). A correlation matrix can be found in Table S1.

**Figure 1:**
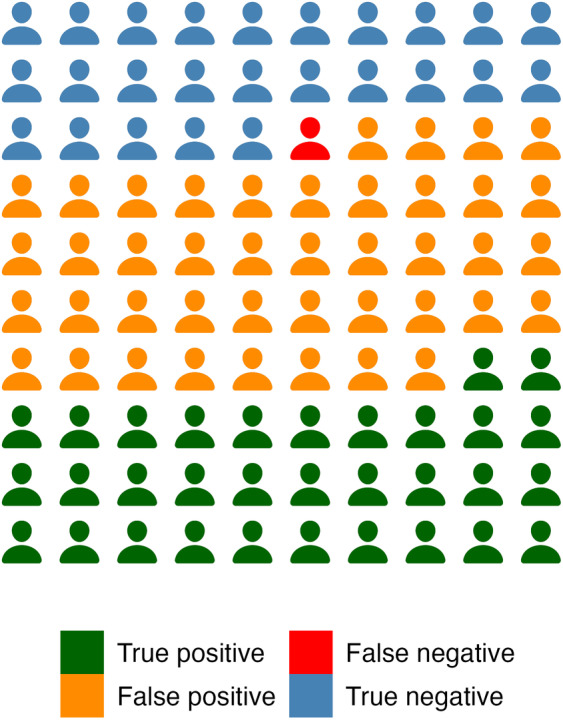
Test properties of the distress thermometer (DT). For clarity, approximate values are used (prevalence 33%, sensitivity 96%, specificity 37%). True positive results: green, false positive: orange, true negative: blue, false negative: red.

In a next step, we used a receiver operating characteristics analysis to determine the optimal cut‐off for the DT to classify PwPD as either being distressed or not distressed. To classify PwPD into these two categories, a total HADS cut‐off score of ≥15 was used, according to previously published literature. This is equal to 33% of PwPD experiencing distress in our study. ROC analysis revealed an area under the curve (AUC) of 0.714. The optimal cut‐off for maximizing sensitivity (97%) and specificity (38%) was set at 4 for the DT. A cut‐off of 5 would have yielded the same value of the Youden's index (0.349), which is a measure of fit for ROC analyses. We chose a cut‐off of 4, because the DT is designed to be a screening tool for distress, hence a high sensitivity and a high negative predictive value is beneficial. The negative predictive value for a cut‐off of 4 is 96%, whereas for a cut‐off of 5 it is 89%. This means that using a cut‐off of 4 for the DT will yield false negative results (ie, patients that are classified as not distressed who actually are in distress) in only 1 out of 100 cases (illustrated in Fig. 1). The trade‐off for this high sensitivity is the higher number of false positive results, resulting in a positive predictive value of 44% (cut‐off 4, ie, people that are determined by the DT to be distressed, who in reality are distressed). However, we believe these properties to be desirable for a screening test. Using the DT cut‐off score of 4, 70% of patients (*N* = 74) were classified as distressed.

No difference was found between male and female patients in exceeding the threshold for distress (Fisher's Exact Test; *p* = 0.64). In addition, PD subtypes did not differ in this regard (*χ* ^2^ test; *p* = 0.34), there was no significant difference in UPDRS III total scores between patients with and without distress (Student's *t*‐test; *p* = 0.19).

To identify underlying contributing factors of distress in PD, we conducted an exploratory factory analysis (EFA), which encompassed the sum scores of all the assessments mentioned above. The first factor consists of the GAD‐7 (factor loading 0.787), HADS‐A (0.954) and FOP‐Q‐SF (0.725) sum scores. The second factor consists of the LQ‐index (−0.842), the HADS‐D (0.675), HHI (−0.608) and UPDRS III (0.657) sum scores. The rotated solution explains 58.4% of the variance in the data set. This is close to the commonly applied 60% cut‐off in social sciences.^26^ The retained factors are highly correlated with each other (*r* = 0.594).

**Figure 2:**
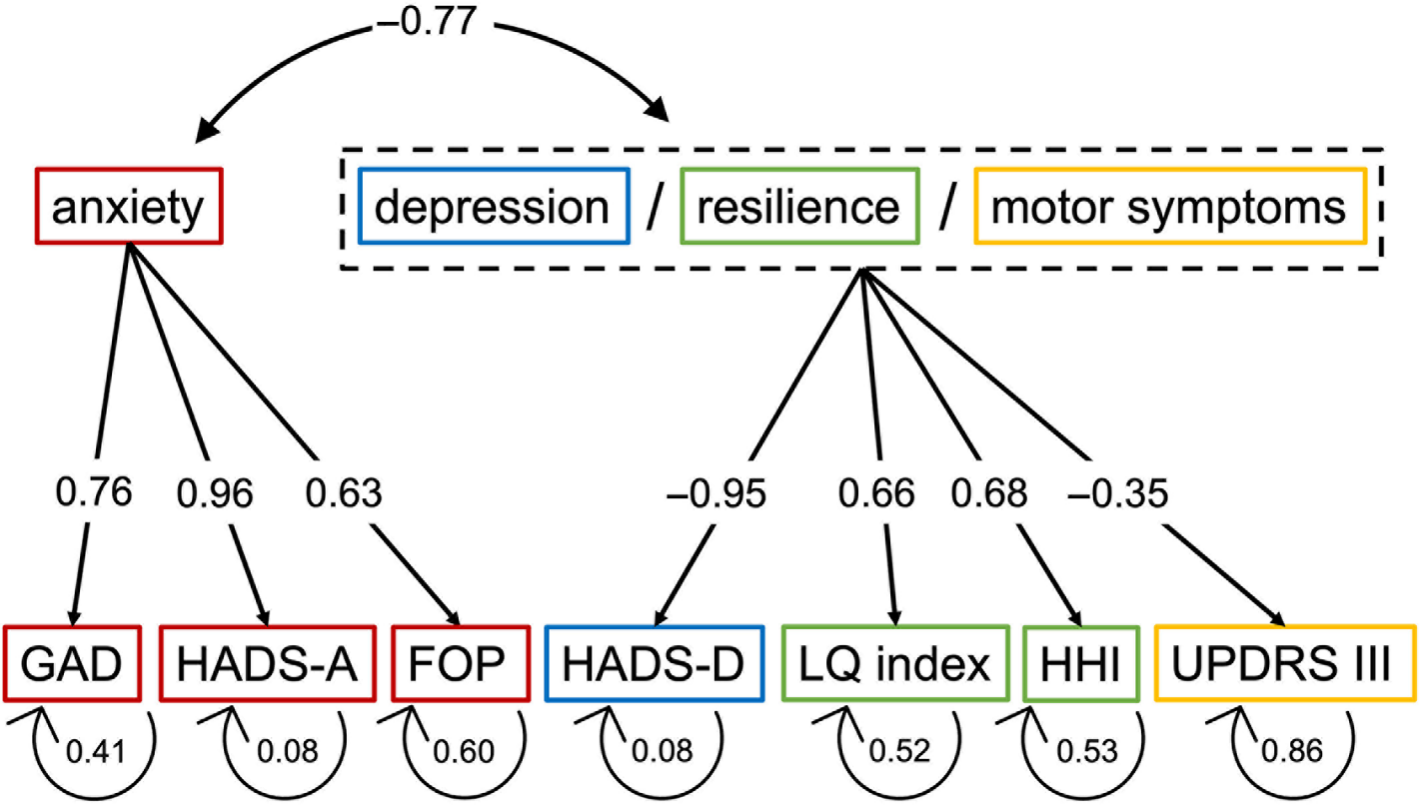
Factor structure of psychosocial distress. Factors identified by exploratory factor analysis. Factor covariances (top arrow), factor loadings (straight arrows) and residual variances (bent arrows) according to the results of a confirmatory factor analysis. FOP, Fear of Progression‐Questionnaire Short Form; GAD, Generalized Anxiety Disorder Scale‐7; HADS‐A, Hospital Anxiety and Depression Scale–Anxiety; HADS‐D, Hospital Anxiety and Depression Scale–Depression; HHI, Herth Hope Index; LQ index, Schedule for the Evaluation of Individual Quality of Life; UPDRS III: Unified Parkinson's Disease Rating Scale part III.

We then conducted a confirmatory factor analysis using the same factors identified by the EFA. For this model, we evaluated further fit indices. A *χ* ^2^ test was significant, so that the null‐hypothesis (perfect model fit) can in theory be rejected, but this test is sensitive to sample size. The Comparative Fit Index showed a value of 0.96, indicating a good fit. Root mean square error of approximation was 0.095, which is close to the recommended value of 0.08. Furthermore, the RMSEA *p*‐value was non‐significant, indicating a well‐fitted model. The standardized root mean square residual was 0.056 (recommended 0.08). Interpreting these indices, one can assume that the factors extracted during exploratory factor analysis make up a model which is well fitted to the data. ^27^ Based on the scales that make up these factors, we termed the first factor “anxiety” and the second factor “depression/resilience/motor symptoms”. This factor structure, its factor covariances, factor loadings and residual variances are displayed in Fig. 2.

In a further step, we trained a random forest regressor to estimate DT values from the variables identified by the factor analyses. This model used 27 trees and two features per split to estimate DT values. It could estimate the DT value with a mean squared error (MSE) of 0.639 and an out‐of‐bag error of 0.78. The random forest regressor trained on the factors identified by factor analysis was able to estimate the DT values from the factors “anxiety” and “depression/resilience/motor symptoms” with a mean absolute error of 0.72 on the 11‐point DT scale. The R^2^ of 0.51 indicates that about half the variance in the dataset is explained by this model.

## Discussion

In this study, we validated the Distress Thermometer as a screening tool for determining distress in people with PD, defined a cut‐off score for the DT and analyzed underlying factors of distress.

As for other diseases, we observed a strong correlation between the DT and the HADS, which confirms the adequacy of this instrument to measure distress in PwPD.

A DT score of 4 was determined to be the optimal cut‐off value for PD using ROC analysis, which is the same value found in oncology and other fields.^7^ With this cut‐off, the DT has a very high sensitivity and thus a high negative predictive value—as expected for a screening test. This means that only about 1 in 100 assessed patients will be wrongly classified as not distressed. Consequently, in case of pathological DT values, the need for psychological or pharmacological treatment should be evaluated. Due to the high sensitivity of this screening tool, there is a relevant amount of false positive results, ie, the actual need for professional psychosocial support is likely to be lower.^28^

The lack of correlation with the H&Y‐stage confirms that disease severity does not necessarily influence distress,^29^ which is similar to findings in patients with brain tumors.^29^ Yet, it should be noted that H&Y‐stages II and III were more common than stages I and IV in the study cohort, and a stronger correlation with disease severity cannot be ruled out in cohorts with a broader range of disease severity. Motor function as measured by the UPDRS III was only weakly correlated with the DT scores, but moderately correlated with measures of depression, QoL and the feeling of hope. Motor function and disease severity alone do not adequately predict QoL in our cohort, as has previously been demonstrated in PD^30^ and other neurodegenerative diseases.^31^, ^32^ Nevertheless, our factor analyses revealed that motor symptoms influence distress to some capacity, albeit to a lesser degree than the other factors in our model. Still, we did not find significant differences concerning motor function between patients with and without distress.

Furthermore, our factor and correlation analyses confirmed that in PwPD, anxiety and depression are important factors in distress,^10^ consistent with findings in other conditions.^3^, ^6^, ^7^ However, the analysis also revealed that distress can be to some degree counterbalanced by feelings of hope (as measured by the HHI) and by a favorable QoL (as measured by the SeiQoL).

Using our model, we could estimate DT values via a random forest regressor with a mean absolute error of 0.72 on an 11‐point scale. This means that this model can accurately predict distress in PD as measured by the DT. On the other hand, this estimate only explains about half of the variance in our dataset. Thus, it represents an oversimplification in and of itself. The PL, which needs to be adapted for the DT in PD, may serve for identification of additional contributing factors in a future validation study.

Similar observations about the relationship between hope, anxiety and depression have been made in other diseases. Strong negative correlations between hope and depressivity have been described in patients with depression and were also apparent in our study cohort.^33^ Furthermore, a study conducted in patients at risk for familiar colorectal cancer showed that hope prevented anxiety and depression 1 year after the disclosure of the results of genetic testing.^34^ Conversely, hopelessness has been shown to predict suicidal ideation and self‐injurious behavior in patients with depression.^35^

The feeling of hope and a favorable QoL in general can be interpreted as an individual's capacity of psychosocial resilience. Resilience has been shown to play a vital role in the mental health and QoL in PwPD.^36^ Our findings indicate that psychosocial resilience is a key preventive factor for distress in PD. Because of this, increasing resilience by means of increasing social support, fostering coping strategies and even physical activity like mind–body exercises may lessen the extent to which PwPD feel distressed.^37^ This extends to the treating physician as well, as the feeling of being in control has been shown to be a relevant factor of resilience in reacting to external stressors (eg, in relation to the COVID‐19 pandemic), underlining the importance of shared decision‐making in PD.^38^ In this regard, patient education programs—which have been shown to be beneficial to QoL in PD^39^—are potentially useful in promoting hope and the feeling of self‐efficacy, since it has been shown that PwPD feeling knowledgeable about PD tend to believe that it is possible to live well with PD.^40^

The findings of this study demonstrate that distress in PD—which is per its definition a multifactorial phenomenon^3^—is distinct from depression, goes beyond anxiety^3^ and requires different treatment strategies. It represents an overarching construct that is relevant in chronic disease in general.^7^, ^28^ For this reason, distress in PwPD should be assessed separately from depression and anxiety. Only assessing these symptoms will not adequately capture distress in PwPD and thus, the need for psychosocial counseling might be underestimated. One third of PwPD are experiencing relevant distress according to their total HADS scores (ie, the true positive rate of the DT results). This is comparable to cancer patients, suggesting that psychosocial support is as necessary in PwPD as in patients with malignant diseases.^41^

There are limitations to this study. (1) We relied on a single center and a relatively small, yet adequate, sample size. (2) Since convenience sampling was applied in this study, PwPD with limited mobility or under palliative care are underrepresented. Ethnic diversities could not be explored, since the population sampled is very homogenous, as is typical for the region of Germany where this study was conducted. This limits the generalizability of our results. (3) Since a low number of patients in our study had a previously diagnosed mild cognitive impairment (MCI) or Parkinson's disease dementia (PDD), the validity of the DT in these conditions could not be explored separately. A future, separate validation of the DT in patients with MCI or PDD would be useful to examine the adequacy of this screening tool in these conditions. (4) We validated the German version of the DT, the English translation of the DT was not validated in this study. However, we believe that the DT is a sufficiently simple screening tool, so that validation across different languages is not necessarily warranted.

In conclusion, the DT is a valid screening tool for distress in PwPD. Its brevity, conciseness and high sensitivity make it ideally suited for clinical practice. There is merit in assessing distress separately from anxiety and depression. Embedding the DT in an automated screening process (e.g. via tablet PC) and its integration into the clinical documentation system could make it readily available to the treating physician.

## Author Roles

(1) Research project: A. Conception, B. Organization, C. Execution; (2) Statistical Analysis: A. Design, B. Execution, C. Review and Critique; (3) Manuscript Preparation: A. Writing of the first draft, B. Review and Critique.

N.S.: 2A, 2B, 2C 3A, 3B.

E.T.: 1A, 1B, 2C, 2A, 2B.

T.F.: 1A, 2A, 2B, 2C, 3B.

A.F.: 2C, 3B.

H.R.: 2C, 3B.

B.F.: 1A, 2B, 2C, 3B.

S.D.S.: 1A, 1B, 1C, 2A, 2B, 2C, 3A, 3B.

## Disclosures

**Ethical Compliance Statement:** All patients gave written informed consent to participate in this study. The study was approved by the ethics committee at Technische Universität Dresden (IRB00001473, EK 37012019) and conducted in accordance with relevant guidelines and regulations. We confirm that all authors have read the Journal's position on issues involved in ethical publication and affirm that this work is consistent with those guidelines.

**Founding sources and Conflict of Interest:** None.

**Financial Disclosures for the previous 12 months:** NS reports no external funding related to the conduct of this study. Outside of the submitted work, he reports travel grants by Abbott, EverPharma and the International Parkinson's Foundation. ET, TF, AF and HR have nothing to report. BF reports no external funding related to the conduct of this study. Outside of the submitted work, he reports grants from the German Research Foundation (DFG) and speaker honoraria from AbbVie, Stadapharm, Desitin, Zambon and Bial. SDS has nothing to report.

## Supporting information


**Figure S1.** Distress thermometer (DT) in its German version.
**Figure S2.** Distress thermometer (DT) in its English version.
**Table S1.** Results of correlation analysis plotted as a matrix. Pearson's r was used for all correlations. Levels of significance are indicated by asterisks; *p < 0.05, **p < 0.01, ***p < 0.001. DT, Distress Thermometer; UPDRS III, Unified Parkinson's Disease Rating Scale part III; total NMS, total non‐motor symptoms as measured by the Non‐Motor Symptoms Questionnaire (NSMQ); LQ index, life quality index as measured by the Schedule for the Evaluation of Individual Quality of Life (SeiQoL); HHI, Herth Hope Scale; PHQ‐9, 9‐question Patient Health Questionnaire; HADS, Hospital Anxiety and Depression Scale; HADS‐A, HADS‐Anxiety subscale; HADS‐D, HADS‐Depression subscale; FOP, Fear of Progression‐Questionnaire; GAD‐7, Generalized Anxiety Disorder 7.
