# Supplementary material for: Validation of the Distress Thermometer as a Screening Tool for Psychosocial Distress and Resilience in Parkinson's Disease
Source: Mov Disord Clin Pract. 2023 Dec 10;11(3):257–64. doi: 10.1002/mdc3.13937 (PMC10928350; doi:10.1002/mdc3.13937)

**Supplemental material**

***Supplemental figure 1.*** Distress thermometer (DT) in its German version

***
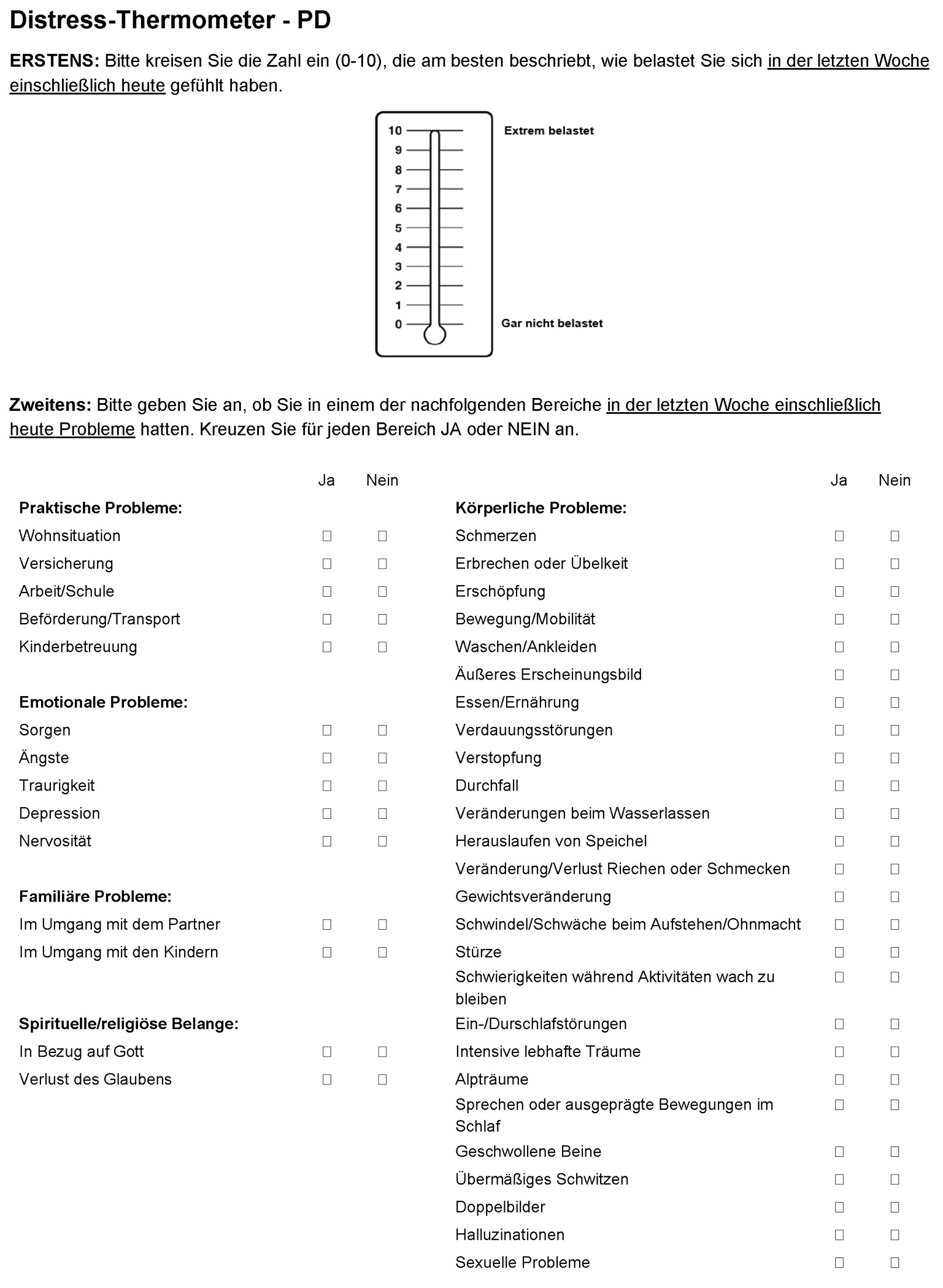
***

***Supplemental figure 2.*** Distress thermometer (DT) in its English version


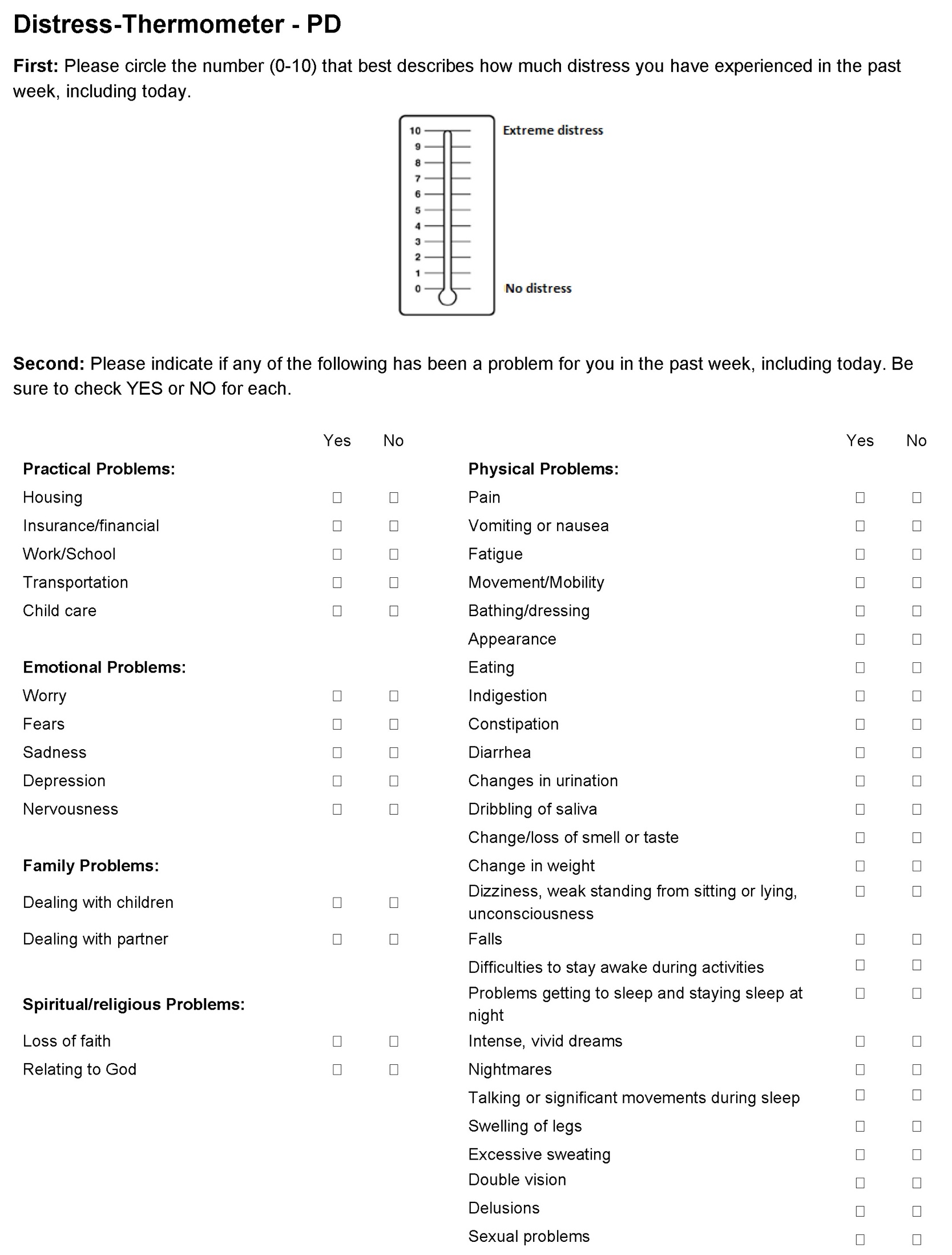


***Supplemental table 1.*** Results of correlation analysis plotted as a matrix. Pearson’s r was used for all correlations. Levels of significance are indicated by asterisks; * p<0.05, ** p<0.01, *** p<0.001. DT: Distress Thermometer; UPDRS III: Unified Parkinson’s Disease Rating Scale part III; total NMS: total non-motor symptoms as measured by the Non-Motor Symptoms Questionnaire (NSMQ); LQ index: life quality index as measured by the Schedule for the Evaluation of Individual Quality of Life (SEIQoL); HHI: Herth Hope Scale; PHQ-9: 9-question Patient Health Questionnaire; HADS: Hospital Anxiety and Depression Scale; HADS-A: HADS-Anxiety subscale; HADS-D: HADS-Depression subscale; FOP: Fear of Progression-Questionnaire; GAD-7: Generalized Anxiety Disorder 7;


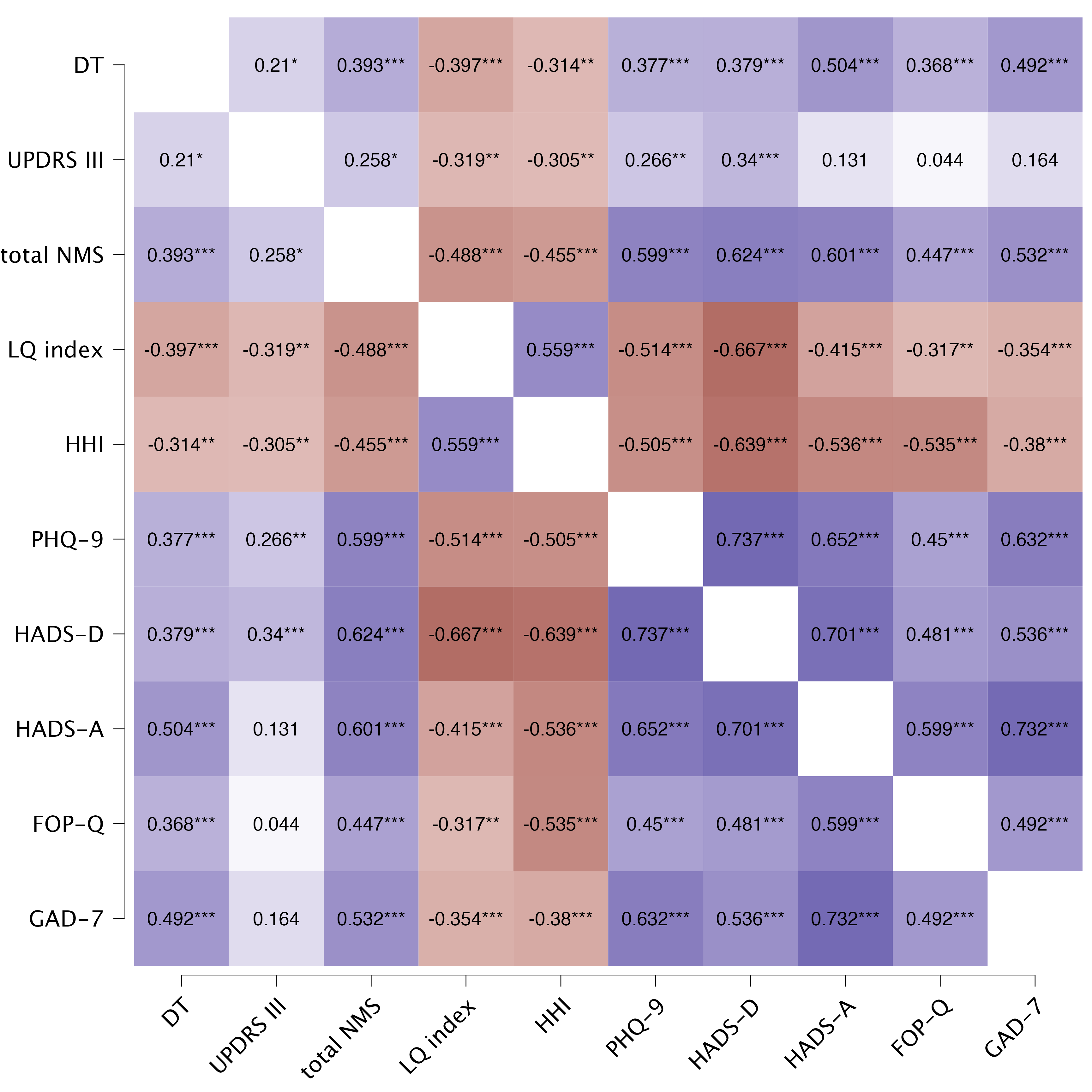

Supplement: Supplementary file 1 — Figure S1. Distress thermometer (DT) in its German version. Figure S2. Distress thermometer (DT) in its English version. Table S1. Results of correlation analysis plotted as a matrix. Pearson's r was used for all correlations. Levels of significance are indicated by asterisks; *p < 0.05, **p < 0.01, ***p < 0.001. DT, Distress Thermometer; UPDRS III, Unified Parkinson's Disease Rating Scale part III; total NMS, total non‐motor symptoms as measured by the Non‐Motor Symptoms Questionnaire (NSMQ); LQ index, life quality index as measured by the Schedule for the Evaluation of Individual Quality of Life (SeiQoL); HHI, Herth Hope Scale; PHQ‐9, 9‐question Patient Health Questionnaire; HADS, Hospital Anxiety and Depression Scale; HADS‐A, HADS‐Anxiety subscale; HADS‐D, HADS‐Depression subscale; FOP, Fear of Progression‐Questionnaire; GAD‐7, Generalized Anxiety Disorder 7. [file MDC3-11-257-s001.docx]
